# The Antimicrobial Activities of Silver Nanoparticles from Aqueous Extract of Grape Seeds against Pathogenic Bacteria and Fungi

**DOI:** 10.3390/molecules26196081

**Published:** 2021-10-08

**Authors:** Fatimah Al-Otibi, Shahad K. Alkhudhair, Raedah I. Alharbi, Abdulaziz A. Al-Askar, Reem M. Aljowaie, Sameha Al-Shehri

**Affiliations:** Department of Botany and Microbiology, College of Science, King Saud University, P.O. Box 22452, Riyadh 11495, Saudi Arabia; sshahadkhaled@gmail.com (S.K.A.); raalharbi@ksu.edu.sa (R.I.A.); aalaskara@ksu.edu.sa (A.A.A.-A.); raljowaie@ksu.edu.sa (R.M.A.); salshihri@ksu.edu.sa (S.A.-S.)

**Keywords:** grape seed extract, sliver nanoparticles, UV spectroscopy, DLS, FTIR, FE-SEM, TEM, GC/MS, antimicrobial activities

## Abstract

Grape seed extract (GSE) is a natural source of polyphenolic compounds and secondary metabolites, which have been tested for their possible antimicrobial activities. In the current study, we tested the antibacterial and antifungal activities of aqueous GSE and the biosynthesized silver nanoparticles loaded with GSE (GSE-AgNPs) against different pathogens. The biosynthesized GSE-AgNPs were assessed by UV spectroscopy, dynamic light scattering (DLS), field emission scanning electron microscopy (FE-SEM), transmission electron microscopy (TEM), Fourier-transform infrared spectroscopy (FTIR), and gas chromatography/mass spectrometry (GC/MS). The antimicrobial activities were assessed against different bacterial and fungal species. DLS analysis showed that GSE-AgNPs had a Z-Average of 91.89 nm while UV spectroscopy showed that GSE-AgNPs had the highest absorbance at a wavelength of ~415 nm. FTIR analysis revealed that both of GSE and GSE-AgNPs consisted of different functional groups, such as hydroxyl, alkenes, alkyne, and aromatic rings. Both FE-SEM and TEM showed that GSE-AgNPs had larger sizes and rough surfaces than GSE and AgNO_3_. The results showed significant antimicrobial activities of GSE-AgNPs against all tested species, unlike GSE, which had weaker and limited effects. More studies are needed to investigate the other antimicrobial activities of GSE.

## 1. Introduction

The use of natural organic materials in the food industry (e.g., plant extracts to replace chemical or synthetic antimicrobials and antioxidants in the treatment of various foodborne pathogens) is trending worldwide [1]. Furthermore, drug resistance and decreased biosafety levels have prompted researchers and microbiologists to seek natural alternatives of known and confirmed antimicrobial characteristics [2]. The usage of plant extracts in folk medicine and pharmaceutics is because they are rich in polyphenols, quinones, flavonoids, alkaloids, and lectins [3].

Grape seed extract (GSE) is rich in polyphenolic compounds and secondary metabolites, which have significant antimicrobial activities against different pathogens, such as Gram-negative and Gram-positive bacteria [4]. The effectiveness of GSE inhibition depends on the concentration of the extract, percentage of phenols, and the type of bacterium [5]. Furthermore, GSE was tested for its possible anti-inflammatory, cardioprotective, chemopreventive, anticarcinogenic, and antioxidant activities, which might be of pharmacological and medical importance [6]. Similar to most natural products, GSE is almost safe, with an intended uptake for up to 11 months in human studies without any significant side effects. Moreover, the food and drug administration (FDA) has generally recognized it as safe (GRAS notice (GRN) no. 658) [7]. This shows that GSE is a promising antimicrobial agent in medical and non-medical studies and applications.

The pH and solubility levels are key regulators of the antimicrobial effects of different plant extracts, which can further affect the sensibility of some microbes to the inhibitory effects [8]. The unique, superior, and indispensable properties of nanomaterials have resulted in emerging innovative nanotechnology being explored in various biological and medical studies [9]. The eco-friendly synthesis of monodispersed nanoparticles using plant extracts (i.e., against various microbial and chronic diseases) has created remarkable advantages in the pharmacological industry [10].

This current study evaluates the inhibitory effects of aqueous GSE against some pathogenic bacteria and fungi, and assesses the antimicrobial activities of GSE green nanoparticles.

## 2. Results and Discussion

### 2.1. The Morphological Characteristics the Biosynthesized GSE-AgNPs

**Figure 1:**
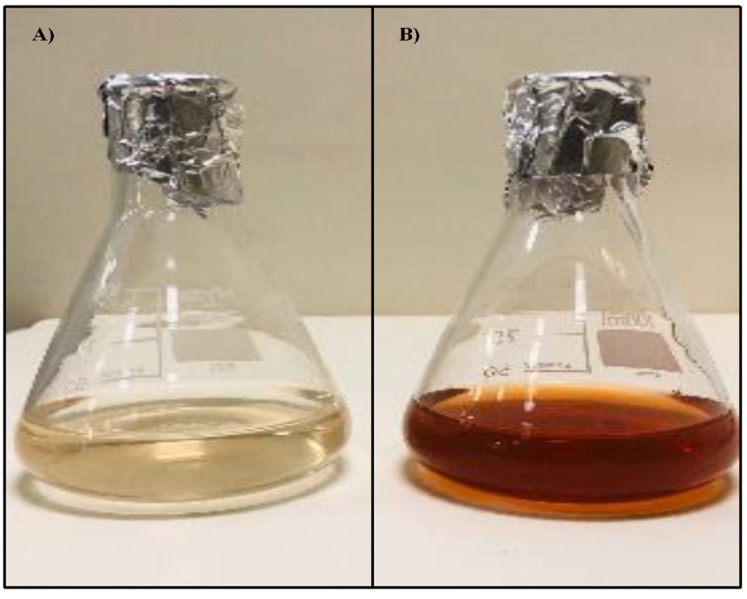
Physical biosynthesis of GSE-AgNPs. GSE-AgNPs were prepared by boiling with an aqueous solution of AgNO_3_ (1 mM), which turned it from light-yellow into brownish-yellow. (**A**) GSE, (**B**) GSE-AgNPs.

One of the most widely-used techniques for the green synthesis of silver nanoparticles is the method described by Xu et al. 2015. The production of AgNPs in a colloidal form depends on the physical reduction of an aqueous solution of silver nitrate (AgNO_3_) [11]. This method has the advantage of a higher purity of synthesized particles due to the absence of a chemical solvent [12]. The usage of a ceramic heater will concentrate the solution by evaporation and induce vibrational motion on the surface plasmon, which will further reduce the silver ions (Ag^+^) (yellowish) and form spherical NPs (brownish) without agglomeration [13]. In the current study, GSE was mixed with an aqueous solution of AgNO_3_ that turned from light yellow to brown (Figure 1), which indicates the formation of GSE-AgNPs. It was reported that GSE contains a great variety of polyphenolic antioxidants, such as proanthocyanidins, which consist of dimers, trimers, tetramers, and oligomers of monomeric catechins [14], which might explain the ability of GSE to induce an efficient reduction of Ag^+^ ions to obtain stable GSE-AgNPs for long-term techniques.

**Figure 2:**
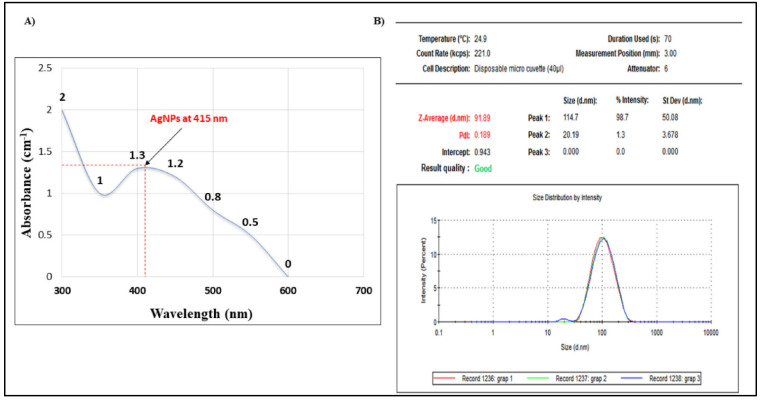
Analysis of the physical properties of GSE-AgNPs. The morphological analysis of the biosynthesised GSE-AgNPs showed their ability to induce higher SPR by UV excitation through either (**A**) UV spectroscopy, or (**B**) the size distribution curve by Zetasizer; three preparations of GSE-AgNPs, at the same concentrations, were used to confirm the results of DLS.

To study the characterization of GSE and the formed nanoparticles, several experiments were performed to investigate the different chemical and morphological changes. The optical properties of GSE-AgNPs are a reflection of their morphological characteristics, such as size, shape, and concentration [15]. A previous study stated that the UV-excitation of colloidal structures of silver nanoparticles can induce a surface plasmon resonance (SPR) with maximum absorbance of 400–500 nm [16]. In the current study, the UV spectroscopy showed that GSE-AgNPs had the highest absorbance broad peak (~1.3 cm^−1^) at the wavelength of ~415 nm (Figure 2A), which reflects the surface plasmon resonance of the synthesized nanoparticles. In accordance with our findings, a previous study showed that the AgNPs of grapefruit (*Vitis vinifera*) extract showed a spectral band at 450 nm, where that higher OD reflected the reduction of the Ag^+^ ions to form the metallic (Ag^0^) [17]. Another study showed that pH induced a shift of the absorbance peaks of AgNPs of different extracts of grape stalk waste from 307 nm in the acidic medium (pH 2), 450 nm at the neutral pH 7 with the Milli-Q water as a solvent, and 470 higher pH, which indicated the stability of aqueous GSE-AgNPs [18]. Another study used two different nanoparticles of silver and iron of the proanthocyanidin compound isolated from grape seeds and showed that the silver nanoparticles had higher absorbance (530 nm) compared to iron (380 nm) [19]. All of these studies suggest that silver nanoparticles could be reduced successfully with GSE aqueous extract using the green physical biosynthesis and that the resulting GSE-AgNPs will have higher stability compared to other solvents, nanoparticles, or chemical synthesis techniques.

The results of the dynamic light scattering (DLS) analysis showed that GSE-AgNPs had the Z-Average of 91.89 nm with a polydispersity index (PDI) value of 0.189 and intercept of 0.943. As shown in Figure 2B, DLS analysis produced the highest peak at 114.7 ± 50.08 nm with 98.7% intensity. In contrast, a previous study showed that the DLS results of synthesized silver nanoparticles of grapefruit extract had a smaller diameter size of 19 nm [17]. Another study showed that the solid lipid nanoparticles loaded with grapeseed or grape skin extracts had mean diameter sizes of 189 ± 2 and 188 ± 18, respectively, compared to the unloaded SLN with 142 ± 10 nm, and that were used in the treatment of human brain-like endothelial cells [20]. Another study showed that SLN particles loaded with grape seed-derived proanthocyanidins had an average diameter size of 243 ± 24 nm with PDI of 0.41–0.51, which successfully increased the ROS production in airway epithelial cells [21]. All of these studies showed that, despite the different sizes of different nanoparticles of grape extracts, all of them were efficient delivery systems. To confirm the morphological characteristics of the biosynthesized nanoparticles, field emission scanning electron microscopy (FE-SEM) and transmission electron microscopy (TEM) were used. Estimations of different particle sizes were measured by ImageJ version 1.51j8 (National Institutes of Health (NIH), Bethesda, MD, USA) https://imagej.nih.gov/ij/ (accessed on 1 May 2021).

**Figure 3:**
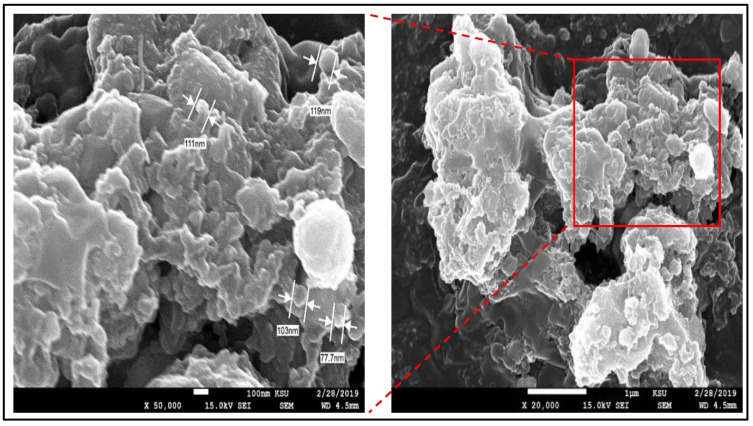
The FE-SEM imaging of GSE-AgNPs. A total of 8 μL were loaded on the 200 mesh grids with a carbon support film of JEOL JEM-2100 microscope. JEM-2100 optic system with 15 kV was used to capture the SEM images at the magnification powers of 20,000× (**right**) and 50,000× (**left**).

**Figure 4:**
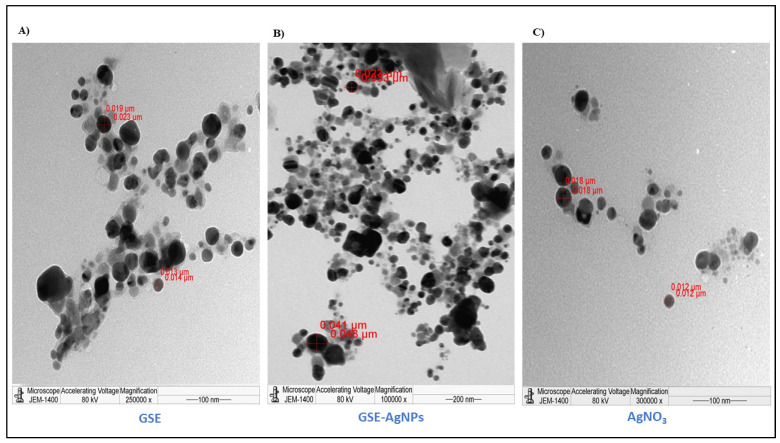
The TEM imaging of GSE-AgNPs. JEM-1011 transmission electron microscope was used to investigate the crystalline structure of (**A**) GSE, (**B**) GSE-AgNPs, and (**C**) AgNO_3_. The diameter sizes of the tested materials were measured by ImageJ software.

The FE-SEM results showed that the GSE-AgNPs had less-smooth surfaces with an average diameter size of 103–119 nm compared to the unloaded nanoparticles of 77.7 nm (Figure 3). However, the results of TEM showed that GSE, AgNO_3_, and GSE-AgNPs had diameter sizes of 13–23 nm, 12–18 nm, and 44–49 nm, respectively, which appeared as spherical dense crystals (Figure 4). These differences in size might be due to the technical differences between the two techniques, where the higher magnification power of TEM (100,000×) recognizes the smaller particles, while the too-low magnification power of SEM (50,000×) will show the particles with wider diameters. Furthermore, SEM is based on using the reflected or knock-off electron beams, which might show wider sizes due to the variable morphologies of the particles where TEM is based on, using the transmitted electrons, which penetrate through the particles to create more accurate images of the scanned particles [22].

Different studies used these two imaging techniques to evaluate the morphological characteristics of GSE nanoparticles. In agreement with our findings, a previous study reported that TEM analysis of AgNPs of grape stalk waste extract had a diameter of 54.3 ± 0.1 nm at pH 6, which decreased with acidic pH 4 to 27.7 ± 0.6 nm [18]. Similarly, another study showed that TEM images of AgNPs loaded with grapefruit extracts had a size diameter of 18–20 nm where the particles appeared as crystalline spheres [17]. In contrast, another study showed that TEM imaging resulted in an average size of 187–191 nm for SLN nanoparticles of grape extracts [20]. Despite different sizes obtained by these techniques, all of them confirmed the synthesis of GSE-AgNPs.

### 2.2. The Chemical Composition Analysis of the Studied Materials

**Figure 5:**
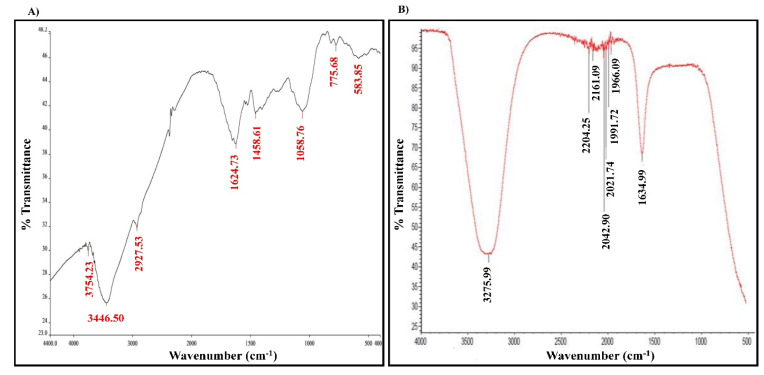
FTIR analysis. Nicolet 6700 FTIR Spectrometer was used to identify the functional groups in (**A**) GSE and (**B**) GSE-AgNPs at the range of 500–4000 cm^−1^.

In addition to the morphological characteristics, it was mandatory to analyze the chemical composition of the studied materials to investigate more about their functional groups. To achieve that purpose, the Fourier-transform infrared spectroscopy (FTIR) and gas chromatography/mass spectrometry technique (GC/MS) were used to analyze the different functional groups or the phenolic constituents of studied materials. FTIR analysis of GSE showed seven distinct functional groups of single bonds (alcohol and aliphatic alkene) and double bonds (olefinic alkene and aromatic rings (polysaccharides). On the other hand, FTIR analysis of GSE-AgNPs resulted in eight functional groups with single bonds (alcohols), double bonds (olefinic alkene, aromatic rings, and nitrogen compounds), and triple bonds (alkynes) (Figure 5, Table 1). The reduction in the number of the functional alcoholic groups from two in GSE to only one in GSE-AgNPs can be explained by the higher boiling point of -OH groups [23]. This increases the ability and suitability of nanoparticles to hydrogen bonding and other chemical reactions, where the biosynthesized GSE-AgNPs are more stable against these reactions [23]. The stronger triple bonds (alkyne groups) in GSE-AgNPs might refer to their higher stability, as well, compared to GSE. Both materials had multiple aromatic compounds, which might be due to the higher polysaccharide content of the seeds. Furthermore, some nitrogen compounds were detected in FTIR analysis of GSE-AgNPs, which might be explained by the reaction of the free NO_3_^−^ ions with other products of the grapeseed extract (Table 1). In agreement with our findings, several studies showed that the FTIR analysis of GSE (water extract) had cis double bond =CH, methylene -CH_2_, aromatic C-C-valence, CH_3_, and CH_2_ aliphatic functional group [19,24].

The GC/MS analysis of GSE showed the presence of five phenolic compounds; 3-hydroxyflavone, anthocyanins, gallic acid, cianidanol, and epicatechin gallate (Table 2). According to the Retention Index (RI) threshold calculations, GSE contained 3-Hydroxyflavone, which was reported to have stronger antimicrobial activity against different bacterial and fungal strains [25]. Moreover, 47.83% RI revealed the presence of anthocyanins, which is a sensitive antimicrobial agent against *Candida albicans*, *Staphylococcus aureus*, *Escherichia coli*, *Enterococcus faecalis*, and *Streptococcus pyogenes* [26,27]. The flavone compounds of gallic acid, cianidanol, and epicatechin gallate had adequate antibacterial activities against *E. coli*, *Pseudomonas aeruginosa*, *S. aureus*, and *Bacillus subtilis*, as well [28].

Several studies reported similar findings for GC/MS analysis of GSE. In the study conducted by Gorodyska et al. 2018, the isopropanol extract of red grape seeds (*Vitis vinifera* L.) contained several phenolic compounds, such as gallic acid, ellagic acid, epicatechin, kaempferol, and myricetin [29]. Another study detected cyanidin, catechin, chlorogenic acid, gallic acid, ellagic acid, epicatechin gallate, and proanthocyanidin B in the methanolic extract of grapeseed [30,31]. All of these characteristics show the importance of GSE as a possible antimicrobial agent.

### 2.3. Antibacterial Activity of GSE and the Biosynthesis of GSE-AgNPs

**Figure 6:**
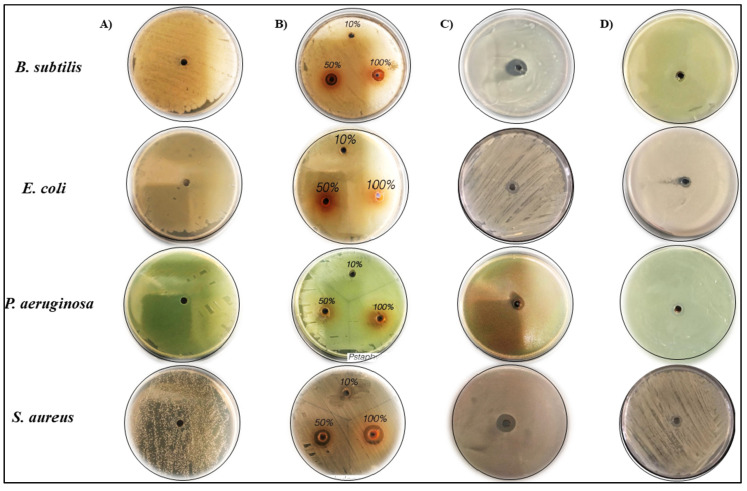
The antibacterial activity of aqueous GSE and the biosynthesized GSE-AgNPs. The bacterial growth was detected by measuring the inhibition zone using the agar disk-diffusion method by the Mueller–Hinton Agar. The plates were treated with (**A**) Milli-Q water as control, (**B**) GSE extract (100%, 50%, & 10%), (**C**) 0.2 mL AgNO_3_, or (**D**) 0.2 mL GSE-AgNPs. The zone of inhibition was measured after seven days ImageJ at the scale of 2.61 pixels/mm.

In the current study, we tested the inhibitory effect of the aqueous GSE and the biosynthesized nanoparticles on the bacterial growth of two Gram-positive strains (*S. aureus* and *B. subtilis*) and two Gram-negative strains (*E. coli* and *P. aeruginosa*), as shown in Figure 6. The treatment with either 50 or 100% of aqueous GSE solution resulted in strong inhibition of *B. subtilis* and *S. aureus*, unlike the other bacterial species, which had not been affected.

On the other hand, the treatment with GSE-AgNPs resulted in stronger inhibition of all species; moreover, it inhibited the bacterial growth of *B. subtilis* and *S. aureus* more than the inhibition induced by GSE alone. Neither water nor AgNO_3_ showed any inhibitory effect on the studied species, which confirms the quality of the experiments. The statistical analysis by one-way ANOVA (Table 3) revealed that treatments with 50% and 100% doses of GSE, and GSE-AgNPs induced inhibition zone diameters of 10.5 ± 0.61, 11 ± 0.44, and 13.5 ± 1 mm, respectively, in the plates of *B. subtilis,* which was significant compared to untreated plates, *p* < 0.001. Similarly, in *S. aureus* plates, the treatments with 50% and 100% doses of GSE, and GSE-AgNPs induced inhibition zone diameters of 13.5 ± 1.61, 15 ± 2, and 15 ± 2 mm, respectively, which was also significant compared to the control, *p* < 0.001. GSE did not induce any significant inhibition of either *E. coli* or *P. aeruginosa* species, while GSE-AgNPs induced significant inhibition compared to the untreated control *p* < 0.001. In all species, treatment with GSE-AgNPs induced stronger inhibition than GSE alone, at all doses.

Several studies reported the strong antibacterial properties of grape products. A previous study showed that GSE induced a growth inhibition of 5–7 log CFU/mL and 2.2–2.6 log CFU/mL against *Campylobacter jejuni* [32] and *Alicyclobacillus acidoterrestris* [33], respectively. In agreement with our findings, a previous study showed that the aqueous extract of *Vitis vinifera* L. seeds induced significant growth inhibition of *B. subtilis* and *S. aureus* but not for *P. aeruginosa* [34]. In contrast, another study showed that only the petroleum ether extract of grape seeds at 20% were effective against *B. subtilis*, *E. coli*, *S. aureus*, and *P. aeruginosa,* while the lower concentrations did not induce any significant growth inhibition [35]. This effect might be explained because the petroleum ether will remove fatty material and resale the active materials in the grape seeds, which will induce a more inhibitory effect. Few studies have shown the significant antibacterial activity of GSE-AgNPs against *B. subtilis* [36,37,38], *E. coli* [36,38], *P*, *aeruginosa* [38], and *S. aureus* [36,39]. It is known that silver ions can stimulate the production of reactive oxygen species (ROS), which increase the oxidative stress and DNA fragmentation in cells [40]. However, lower concentrations of AgNO_3_ (<5 mM) were found to induce no DNA fragmentation [41], which explains its null effect in the current study as we used only 0.2 mM. Our results, in combination with the previous studies, suggested GSE-AgNPs as a possible bactericidal agent against both Gram-positive and Gram-negative species.

### 2.4. Antifungal Activity of GSE and the Biosynthesis of GSE-AgNPs

**Figure 7:**
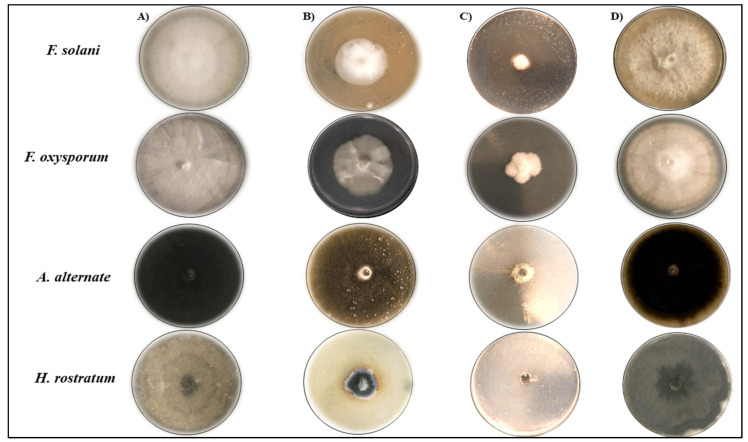
The antifungal activity of aqueous GSE and the biosynthesized GSE-AgNPs. The mycelial growth was measured by the agar well diffusion method using potato dextrose agar (PDA) media. The plates were treated with either (**A**) Milli-Q water as control, (**B**) GSE extract (100%), (**C**) 0.2 mL AgNO_3_, or (**D**) 0.2 mL GSE-AgNPs. The mycelial growth measuring of the colony diameter after seven days. The mycelial growth was measured by ImageJ at the scale of 2.61 pixels/mm.

The effect of aqueous GSE on the mycelial growth of were *Fusarium solani*, *Fusarium oxysporum*, *Helminthosporium rostratum*, and *Alternaria alternata.* The strains were tested by the agar well diffusion method (Figure 7). The results showed that GSE had significant reduction in the growth of all species, except for *A. alternata*, while the slowest growth was for *H. rostratum* by 22.75 ± 0.2 mm compared to the control 87.19 ± 0.07 mm, *p* < 0.001. Similarly, treatment with GSE-AgNPs induced stronger mycelial growth inhibition of all spices than GSE and AgNO_3_. The maximum inhibitory effect was for *H. rostratum* by 9.11 ± 0.03 mm compared to control at 87.19 ± 0.07 mm, *p* < 0.001 (Table 4).

Several studies had evaluated the antifungal activity of GSE, particularly against *Candida* spp. In the study conducted by Eslami et al. 2017, the minimum inhibitory concentration (MIC) of GSE against *Candida glabrata* and *Candida krusei* was 50 µg/mL, which showed significant inhibition compared to the control [42]. Another study showed that GSE at doses of 6–20 mg/L had antifungal activity against *C. albicans*, as well [43]. In accordance with our findings, a previous study showed that the ethanolic extract of grapefruit tendrils had a significant fungicidal effect against different *Fusarium* species such as *F. oxysporum*, *F. culmorum*, *F. solani*, *F. coeruleum*, *F. sporotrichioides*, *F. verticillioides*, and *F. tabacinum,* while another species, *Rhizoctonia solani,* showed significant resistance [44]. Another study showed that polymeric proanthocyanidins isolated from grape seeds by ethanolic extraction had significant antifungal activity against *Botrytis cinerea* by inhibiting the spore germination [45]. Another study showed that the lonely application of GSE did not induce any inhibition of the mycelial growth of *A. alternata* [46]. To our knowledge, this is the first study that reported the antifungal activity of aqueous GSE against *H. rostratum* fungus.

As shown in Figure 7 and Table 4, the biosynthesized nanoparticles almost did not allow the mycelial growth of *H. rostratum* (<10%) while *F. solani* and *A. alternata* had minimum and limited growths of about 20% and 17% compared to the untreated plates. The minimal fungicidal effects of either GSE or GSE-AgNPs were for *F. oxysporum* that grow up to 25% of the untreated fungus. Noticeably, even with limited effect, the growth *of F. oxysporum* was not semi-circular as in the case of the untreated control, but it looks more like a condensed amoeboid-shape, which might suggest weak resistance of the fungal species to GSE-AgNPs. Despite limited studies that demonstrated the antifungal activity of GSE-AgNPs, some studies showed similar effects of other nanoparticles. In the study conducted by Sagana et al. 2020, the zinc oxide nanoparticles of aqueous GSE induced a growth inhibition to 16 mm of *C. albicans* 24 h post-treatment [47]. Similarly, another study showed that titanium oxide nanoparticles of aqueous GSE reduced the growth of *C. albicans* to 12 mm compared to the control [48]. All of these studies, in addition to our findings, highlight the antimicrobial activities of GSE against different pathogenic bacteria and fungi.

## 3. Materials and Methods

### 3.1. Chemicals and Reagents

Silver nitrate (AgNO_3_) and all other chemicals for nanoparticle preparation were purchased from Sigma-Aldrich (Sigma-Aldrich Chemie GmbH, Taufkirchen, Germany). The materials for bacterial and fungal cultures were purchased from Thermo Fisher Scientific (Thermo Fisher Scientific, Waltham, MA, USA).

### 3.2. Preparation of Aqueous GSE

The red grape was purchased from a local market in Saudi Arabia and classified at the department of Botany and Microbiology, Faculty of Science, King Saud University, Riyadh, Saudi Arabia. The fruit diameter was about 6 mm, reddish with pale wax bloom. It was classification as *Vitis vinifera* L., a member of the Vitaceae or Grape family. The seeds were collected, gently washed, air dried, ground by an electric miller, and stored at room temperature (25 °C) until use. An amount of 20 g of the powder was dissolved into 200 mL of ultrapure water, vortexed, and boiled for 15 min. After cooling, the extract was filtered, centrifuged, and the supernatant was collected. The supernatant was yellowish-brown and stored at 4 °C for future use. 

### 3.3. Microorganisms

Both of the previously identified bacterial and fungal strains were obtained from the department of Plant Protection, College of Food and Agricultural Sciences, King Saud University. Four bacterial strains were used for the preliminary studies, *S. aureus*, *E. coli*, *B. subtilis*, and *P. aeruginosa.* The fungal strains were *F. solani*, *F. oxysporum*, *H. rostratum*, and *A. alternata.*

### 3.4. Bio Synthesis of Silver Nanoparticles of GSE

The preparation of AgNO_3_ loaded with aqueous GSE (GSE-AgNPs) was described by Xu et al. 2015 [11]. Briefly, 20 mL of 1 mM aqueous solution of AgNO_3_ were mixed with 1 mL (200 mg/mL) of GSE extract and boiled at 95 °C for 10 min. The new solution of synthesized GSE-AgNPs turned into lighter brown as compared to the light-yellow un-boiled solution of GSE.

### 3.5. Characterization of Synthesized GSE-AgNPs

#### 3.5.1. UV Spectroscopy

UV-visible spectrophotometer (Shimadzu, Tokyo, Japan) was used for the characterization of GSE-AgNPs. The reduction of pure Ag^+^ ions was checked at 200–800 nm by UV-2450 double-beam according to the manufacturer’s instructions, as described previously [49]. The experiment was performed in triplicates.

#### 3.5.2. Dynamic Light Scattering (DLS) Analysis

To measure the stability of the colloidal nanoparticles, it is important to measure their effective surface charge or zeta potential, which reflects their surface energy [50]. To detect the particle size distribution, it is important to use the DLS technique, which will calculate the PDI and Z-Average, which refers to the width of the overall distribution and the size distribution by intensity of the specific particle, respectively [51]. In the current study, DLS was used to measure the PDI and Z-Average by the Zetasizer (Malvern Panalytical, Malvern, UK), according to the manufacturer’s instructions.

#### 3.5.3. Field Emission Scanning Electron Microscopy (FE-SEM)

The FE-SEM technique by JEOL JEM-2100 microscope (JEOL, Peabody, MA, USA) was used to investigate the external morphology and physical characteristics of the synthesized nanoparticles, as described previously [52]. Briefly, a drop of nanoparticle suspensions with a volume of 8 μL was placed onto 200 mesh grids with a carbon support film (Agar Scientific, London, UK), rinsed with ethanol and air-dried. Then, the sample was fixed on an appropriate SEM holder and the images were taken at an accelerating voltage of 15 kV using JEM-2100 optic system (JEOL, Peabody, MA, USA). The experiment was performed in triplicates.

#### 3.5.4. Transmission Electron Microscopy (TEM)

The TEM technique by JEM-1011 transmission electron microscope (JEOL Ltd. Inc., Tokyo, Japan) was used to confirm the crystalline structure of the synthesized nanoparticles. The preparation and processing of slides were according to the manufacturer’s instructions, as described previously [53].

#### 3.5.5. Fourier-Transform Infrared Spectroscopy (FTIR)

FTIR analysis is a powerful analytical technique that is used for the identification (or confirmation) of any unknown material. It works through the radiation of the sample by an infrared radiation of 10,000 to 100 cm^−1^, which causes vibration (or rotation) that can be further detected on a pyroelectric detector at the range of 400–4000/cm. The resulting spectrum is unique to each material and is represented as a number of particular descending peaks of a particular wavenumber, which represent specific functional groups [54]. In the current study, FTIR spectroscopy was used to analyze the components of the newly synthetized GSE-AgNPs to confirm its composition. A dedicated FTIR spectrometer (Nicolet 6700 FTIR Spectrometer, Waltham, MA, USA) at the range of 500–4000/cm was used. The experiment was performed in triplicates.

#### 3.5.6. Gas Chromatography/Mass Spectrometry Technique (GC/MS)

The GC/MS analysis was performed by using a dedicated thermo-gas chromatograph/mass spectrometer (model Shimadzu 2010) equipped with Rtx-5MS capillary column (30 m long, 0.25 mm in diameter, film thickness of 0.25 μm). The carrier gas was helium and the maximum usable temperature was 280 °C. The data were analyzed using the libraries of National Institute of Standards and Technology (NIST) database https://www.nist.gov/ (accessed on 20 May 2021) and Wiley Registry of Mass Spectral Data https://sciencesolutions.wiley.com/solutions/technique/gc-ms/wiley-registry-of-mass-spectral-data-12th-edition/ (accessed on 20 May 2021). The experiment was performed in triplicates.

### 3.6. Determination Antimicrobial Activity

#### 3.6.1. Determination of Antibacterial Activity

The antibacterial activity was evaluated by measuring the zone of inhibition for each bacterial culture by the agar disk-diffusion method, as described previously [55]. Briefly, the bacterial strains were cultured on the Mueller–Hinton Agar (MHA) for 24 h at 37 °C, then two colonies of each plate were transferred to a tube of 10 mL distilled water and mixed thoroughly to maintain uniform distribution. Using sterile swabs, 0.2 mL of bacteria strain (2.5 × 10^5^ CFU/mL) was swabbed uniformly onto individual MHA plate and allowed to dry for ten min. Four plates were prepared for each bacterium to test GSE, AgNO_3_, GSE-AgNPs, and one plate for distilled water to be used as negative control, separately.

The disk-diffusion method was applied by forming of adequately spaced wells (holes) of 4 mm diameter at the culture agar surface using a sterile metal cork borer. For control plates, a negative control was used. To test GSE, three wells were formed, supplied with 0.2 mL of three different concentration of the GSE extract (100%, 50%, & 10%). For AgNO_3_, GSE-AgNPs, and control plates, a single well/plate was formed and treated with 0.2 mL of either water, AgNO_3_, or GSE-AgNPs. All plates were kept under aseptic conditions, at room temperature, for one hour, to allow the agents to diffuse into agar medium. Subsequently, the plates were incubated for 24 h at 37 °C. At the end of the incubation period, the inhibition zones, the area surrounding the hole with no growth of inoculated microorganisms, were measured to the nearest millimeter, as described before [56]. The zone of inhibition was measured by ImageJ version 1.51j8 (National Institutes of Health (NIH), Bethesda, MD, USA) https://imagej.nih.gov/ij/ (accessed on 1 May 2021) at the scale of 2.61 pixels/mm. All experiments were performed in triplicates.

#### 3.6.2. Determination of Antifungal Activity

The antifungal activity was assessed by the agar well diffusion method described by Daoud et al. 2019, with slight modification [57]. Briefly, potato dextrose agar (PDA) media (20 gm dextrose, 15 gm agar, 4 gm potato starch, 40 mg chlortetracycline, 25 mg chloramphenicol, and 1.4 gm tartaric acid) was prepared by boiling for 15 min at 121 °C in one liter of distilled water (pH 3.5 at 25 °C) with mixing to dissolve. The mixture was cooled to 55 to 60 °C followed by mixing with either GSE, AgNO_3_, GSE-AgNPs, or distilled water (negative control), then an appropriate amount from each PDA/antifungal agent was poured in a separate Petri dish and kept in sterile atmosphere until solidification. Later, a flint hole of 1 cm diameter was formed by a sterile metal cork borer at the center of the plate, then the fungus strains were added by direct plating. The mycelial growth inhibition was determined by measuring of the colony’s diameter after seven days. The mycelial growth was measured by ImageJ version 1.51j8 (National Institutes of Health (NIH), MD, United States) https://imagej.nih.gov/ij/ (accessed on 1 May 2021) at the scale of 2.61 pixels/mm. All experiments were performed in triplicates.

### 3.7. Statistical Analysis

The statistical analysis was performed by dedicated software (Minitab 2018, State College, PA, USA). Means and standard deviations were calculated for all quantitative data. One-way ANOVA was used to assess the significance levels of results at *p* < 0.05.

## 4. Conclusions

The current study showed the strong antimicrobial activities of aqueous GSE and the biosynthesized GSE-AgNPs against different Gram-positive, Gram-negative, and fungi species. The novelty of this work is that it is the first reporting/demonstration of antifungal activity of GSE-AgNPs and of the fungicidal activity of GSE or GSE-AgNPs against the red spot fungus *H. rostratum*. However, more studies are needed to investigate and demonstrate the antimicrobial activities of the most active constituents of grape seed and other grape-products.

## Figures and Tables

**Table 1:** The FTIR analysis of the functional groups in the GSE and GSE-AgNPs.

Tested Material	Absorption (cm^−1^)	Appearance	Group	Compound Class
**GSE**	3754	Medium, sharp	O-H stretching	Alcohol
	3446	Strong, broad	Dimeric O-H stretch	
	2927	Strong, broad	Methylene C-H asymmetric	Aliphatic alkene
	1624	Strong	Skeletal C-C vibrations (methyne)	
	775	Medium	Alkenyl C=C stretch	Olefinic alkene
	1458	Medium	C=C-C aromatic ring stretch	Aromatic ring
	1058	Medium	Aromatic C-H in-plane bend	
**GSE-AgNPs**	3276	Strong, broad	Hydroxy group, H-bonded OH stretch	Alcohol
	2204	Weak	C≡C medial alkyne (disubstituted)	Alkyne
	2161	Weak	C≡C terminal alkyne (monosubstituted)	
	1992/1966	Weak	Aromatic combination bands	Aromatic ring
	2043/2022	Weak	Transition metal carbonyls, cyanide, thiocyanate, or isothiocyanate (-NCS)	Carbonyl or inorganic
	1634	Medium	Alkenyl C=C stretch	Olefinic alkene

**Table 2:** GC/MS analysis of GSE phenolic constituents.

Phenolic Compound	Formula	Chemical Structure	Molecular Weight	MS Fragments (m/z)	RI%
**3-Hydroxyflavone**	**C_15_H_10_O_3_**	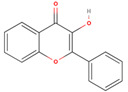	238.242	238.25	74.80
**Anthocyanins**	**C_15_H_11_O^+^**	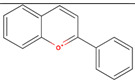	207.252	207.00	47.83
**Gallic acid**	**C_7_H_6_O_5_**	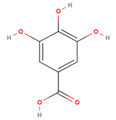	170.12	170.00	29.81
**Cianidanol**	**C_15_H_14_O_6_**	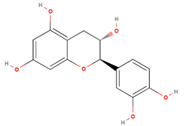	290.271	290.00	6.50
**Epicatechin gallate**	**C_22_H_18_O_10_**	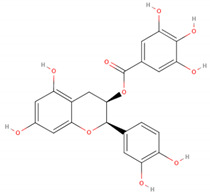	442.376	442.00	1.12

RI: Retention index.

**Table 3:** The antibacterial effect of GSE and biosynthesized nanoparticles by measuring the inhibition zone diameter (mm).

Strains		Control	10%GSE	50%GSE	100%GSE	GSE-AgNPs	AgNO_3_
*B. subtilis*	Mean ± SD	0.07 ± 0.06	0.27 ± 0.21	10.5 ± 0.61	11 ± 0.44	13.5 ± 1	0.5 ± 0.1
	*p*-value	1	0.645	1.13 × 10^−11^ *	7.02 × 10^−12^ *	6.13 × 10^−13^ *	0.327
*E. coli*	Mean ± SD	0.07 ± 0.12	0.2 ± 0.1	0.3 ± 0.17	0.5 ± 0.1	12.5 ± 1	0.7 ± 0.1
	*p*-value	1	0.706	0.512	0.233	1.35 × 10^−13^ *	0.092
*P. aeruginosa*	Mean ± SD	0.07 ± 0.06	0.07 ± 0.06	0.5 ± 0.1	0.7 ± 0.1	13.5 ± 1	0.1 ± 0.1
	*p*-value	1	1	0.226	0.087	4.37 × 10^−14^ *	0.923
*S. aureus*	Mean ± SD	0.03 ± 0.06	0.17 ± 0.16	13.5 ± 1.61	15 ± 2	15 ± 2	0.6 ± 0.1
	*p*-value	1	0.904	3.37 × 10^−8^ *	1.03 × 10^−10^ *	1.03 × 10^−10^ *	0.612

* *p*-value < 0.05 compared to control. SD: standard deviation.

**Table 4:** GSE and biosynthesized nanoparticles affect the mycelial growth (mm) of the studied fungi.

Strains		Control	GSE (100%)	GSE-AgNPs (0.2 mL)	AgNO_3_ (0.2 mL)
*F. solani*	Mean ± SD	88.23 ± 0.06	37.5 ± 0.26	17.52 ± 0.03	88.15 ± 0.1
	*p*-value	1	9.96 × 10^−20^ *	1.21 × 10^−37^ *	0.999
*F. oxysporum*	Mean ± SD	88.39 ± 0.15	67.5 ± 0.2	22.53 ± 0.11	88.59 ± 0.11
	*p*-value	1	6.08 × 10^−4^ *	1.08 × 10^−32^ *	0.957
*A. alternata*	Mean ± SD	88.51 ± 0.11	84.91 ± 0.18	14.49 ± 0.15	88.43 ± 0.09
	*p*-value	1	0.459	4.72 × 10^−41^ *	0.997
*H. rostratum*	Mean ± SD	88.19 ± 0.07	22.75 ± 0.2	9.11 ± 0.03	88.09 ± 0.13
	*p*-value	1	2.33 × 10^−32^ *	6.39 × 10^−47^ *	0.991

* *p*-value < 0.05. SD: Standard Deviation.

## Data Availability

All data presented in this study are available within the current article. All statistical analysis results and raw data are available upon request from the corresponding author.

## Sample Availability

Samples of the GSE and GSE-AgNPs compounds are available from the authors.

## References

[1] Hintz T., Matthews K.K., Di R. (2015). The Use of Plant Antimicrobial Compounds for Food Preservation. Biomed Res. Int..

[2] Hashempour-Baltork F., Hosseini H., Shojaee-Aliabadi S., Torbati M., Alizadeh A.M., Alizadeh M. (2019). Drug Resistance and the Prevention Strategies in Food Borne Bacteria: An Update Review. Adv. Pharm. Bull..

[3] Othman L., Sleiman A., Abdel-Massih R.M. (2019). Antimicrobial Activity of Polyphenols and Alkaloids in Middle Eastern Plants. Front. Microbiol..

[4] Memar M.Y., Adibkia K., Farajnia S., Kafil H.S., Yekani M., Alizadeh N., Ghotaslou R. (2019). The grape seed extract: A natural antimicrobial agent against different pathogens. Rev. Med. Microbiol..

[5] Perumalla A.V.S., Hettiarachchy N.S. (2011). Green tea and grape seed extracts—Potential applications in food safety and quality. Food Res. Int..

[6] Mahmoud Y.I. (2013). Grape seed extract neutralizes the effects of Cerastes cerastes cerastes post-synaptic neurotoxin in mouse diaphragm. Micron.

[7] GRAS Notice 658, Grapefruit Extract—FDA: Notice to the US Food and Drug Administration That the Use of Vancitrix™, a Glycerin Citrus Extract, Is Generally Recognized as Safe. https://www.fda.gov/food/generally-recognized-safe-gras/gras-notice-inventory.

[8] Ramazanzadeh B., Jahanbin A., Yaghoubi M., Shahtahmassbi N., Ghazvini K., Shakeri M., Shafaee H. (2015). Comparison of Antibacterial Effects of ZnO and CuO Nanoparticles Coated Brackets against *Streptococcus* Mutans. J. Dent..

[9] Wang L., Hu C., Shao L. (2017). The antimicrobial activity of nanoparticles: Present situation and prospects for the future. Int. J. Nanomed..

[10] Kuppusamy P., Yusoff M.M., Maniam G.P., Govindan N. (2016). Biosynthesis of metallic nanoparticles using plant derivatives and their new avenues in pharmacological applications—An updated report. Saudi Pharm. J..

[11] Xu H., Wang L., Su H., Gu L., Han T., Meng F., Liu C. (2015). Making Good Use of Food Wastes: Green Synthesis of Highly Stabilized Silver Nanoparticles from Grape Seed Extract and Their Antimicrobial Activity. Food Biophys..

[12] Ji J.H., Jung J.H., Yu I.J., Kim S.S. (2007). Long-term stability characteristics of metal nanoparticle generator using small ceramic heater for inhalation toxicity studies. Inhal. Toxicol..

[13] Iravani S., Korbekandi H., Mirmohammadi S.V., Zolfaghari B. (2014). Synthesis of silver nanoparticles: Chemical, physical and biological methods. Res. Pharm. Sci..

[14] Shi J., Yu J., Pohorly J.E., Kakuda Y. (2003). Polyphenolics in grape seeds-biochemistry and functionality. J. Med. Food.

[15] Zhang X.F., Liu Z.G., Shen W., Gurunathan S. (2016). Silver Nanoparticles: Synthesis, Characterization, Properties, Applications, and Therapeutic Approaches. Int. J. Mol. Sci..

[16] François E.M., Marcelle L.S., Cecile O.E., Agnes A.N., Djiopang Y.S., Fanny A.E.M., Lidwine N., Harouna M., Emmanuel M.M. (2016). Unexplored vegetal green synthesis of silver nanoparticles: A preliminary study with *Corchorus olitorus* Linn and *Ipomea batatas* (L.) Lam. Afr. J. Biotechnol..

[17] Roy K., Biswas S., Banerjee P.C. (2013). Synthesis of Silver Nanoparticles by Using Grape (*Vitis vinifera*) Fruit Extract: Characterization of the Particles and Study of Antibacterial Activity. Res. J. Pharm. Biol. Chem. Sci..

[18] Bastos-Arrieta J., Florido A., Pérez-Ràfols C., Serrano N., Fiol N., Poch J., Villaescusa I. (2018). Green Synthesis of Ag Nanoparticles Using Grape Stalk Waste Extract for the Modification of Screen-Printed Electrodes. Nanomaterials.

[19] Shejawal K.P., Randive D.S., Bhinge S.D., Bhutkar M.A., Wadkar G.H., Jadhav N.R. (2020). Green synthesis of silver and iron nanoparticles of isolated proanthocyanidin: Its characterization, antioxidant, antimicrobial, and cytotoxic activities against COLO320DM and HT29. J. Genet. Eng. Biotechnol..

[20] Loureiro J.A., Andrade S., Duarte A., Neves A.R., Queiroz J.F., Nunes C., Sevin E., Fenart L., Gosselet F., Coelho M.A. (2017). Resveratrol and Grape Extract-loaded Solid Lipid Nanoparticles for the Treatment of Alzheimer’s Disease. Molecules.

[21] Castellani S., Trapani A., Spagnoletta A., di Toma L., Magrone T., Di Gioia S., Mandracchia D., Trapani G., Jirillo E., Conese M. (2018). Nanoparticle delivery of grape seed-derived proanthocyanidins to airway epithelial cells dampens oxidative stress and inflammation. J. Transl. Med..

[22] Brodusch N., Brahimi S.V., Barbosa De Melo E., Song J., Yue S., Piché N., Gauvin R. (2021). Scanning Electron Microscopy versus Transmission Electron Microscopy for Material Characterization: A Comparative Study on High-Strength Steels. Scanning.

[23] Hadjiivanov K., Jentoft F.C. (2014). Chapter Two—Identification and Characterization of Surface Hydroxyl Groups by Infrared Spectroscopy. Advances in Catalysis.

[24] Mohansrinivasan V., Devi S.C., Meenakshi D., Biswas A., Naine J. (2015). Exploring the Anticancer Activity of Grape Seed Extract on Skin Cancer Cell Lines A431. Braz. Arch. Biol. Technol..

[25] Naik K.K., Thangavel S., Alam A., Kumar S. (2017). Flavone Analogues as Antimicrobial Agents. Recent Pat. Inflamm. Allergy Drug Discov..

[26] Salamon I., Şimşek Sezer E.N., Kryvtsova M., Labun P. (2021). Antiproliferative and Antimicrobial Activity of Anthocyanins from Berry Fruits after Their Isolation and Freeze-Drying. Appl. Sci..

[27] Mahmad N., Taha R.M., Othman R., Abdullah S., Anuar N., Elias H., Rawi N. (2018). Anthocyanin as potential source for antimicrobial activity in *Clitoria ternatea* L. and *Dioscorea alata* L.. Pigment Resin Technol..

[28] Li Y., Xia H., Wu M., Wang J., Lu X., Wei S., Li K., Wang L., Wang R., Zhao P. (2017). Evaluation of the Antibacterial Effects of Flavonoid Combination from the Leaves of *Dracontomelon dao* by Microcalorimetry and the Quadratic Rotary Combination Design. Front. Pharmacol..

[29] Gorodyska O., Grevtseva N., Samokhvalova O., Gubsky S. (2018). Determination of the cemical composition of grape seed powders by GC-MS analysis. EUREKA Life Sci..

[30] Aybastıer Ö., Dawbaa S., Demir C. (2018). Investigation of antioxidant ability of grape seeds extract to prevent oxidatively induced DNA damage by gas chromatography-tandem mass spectrometry. J. Chromatogr. B.

[31] Lin L.Z., Sun J., Chen P., Monagas M.J., Harnly J.M. (2014). UHPLC-PDA-ESI/HRMSn profiling method to identify and quantify oligomeric proanthocyanidins in plant products. J. Agric. Food Chem..

[32] Silvan J.M., Mingo E., Hidalgo M., de Pascual-Teresa S., Carrascosa A., Martínez-Rodríguez A. (2013). Antibacterial activity of a grape seed extract and its fractions against *Campylobacter* spp.. Food Control.

[33] Molva E., Baysal A.H. (2015). Antimicrobial activity of grape seed extract on *Alicyclobacillus acidoterrestris* DSM 3922 vegetative cells and spores in apple juice. LWT Food Sci. Technol..

[34] Alkhulaifi M., Alfarraj D., Moubayed N. (2017). In vitro Antibacterial Activity of Red Grape Seed Extracts on some Important Human Pathogenic Bacteria. J. Adv. Microbiol..

[35] Baydar N.G., Özkan G., Sağdiç O. (2004). Total phenolic contents and antibacterial activities of grape (*Vitis vinifera* L.) extracts. Food Control.

[36] Han H.W., Kwak J.H., Jang T.S., Knowles J.C., Kim H.W., Lee H.H., Lee J.H. (2021). Grapefruit Seed Extract as a Natural Derived Antibacterial Substance against Multidrug-Resistant Bacteria. Antibiotics.

[37] Asaduzzaman A.K.M., Chun B., Kabir S.R. (2016). *Vitis vinifera* Assisted Silver Nanoparticles with Antibacterial and Antiproliferative Activity against Ehrlich Ascites Carcinoma Cells. J. Nanopart..

[38] Ramesh G., Joshi P.S., Packiyam J.M., Jayanna S.K. (2017). Biosynthesis and characterization of Silver Nanoparticles from Grape (*Vitis vinifera*) seeds and study on antibacterial activity. Int. J. Curr. Res. Chem. Pharm. Sci..

[39] Shrestha B., Theerathavaj M.L., Thaweboon S., Thaweboon B. (2012). In vitro antimicrobial effects of grape seed extract on peri-implantitis microflora in craniofacial implants. Asian Pac. J. Trop. Biomed..

[40] Matsumura Y., Yoshikata K., Kunisaki S., Tsuchido T. (2003). Mode of bactericidal action of silver zeolite and its comparison with that of silver nitrate. Appl. Environ. Microbiol..

[41] Kumar Pandian S.R., Deepak V., Kalishwaralal K., Viswanathan P., Gurunathan S. (2010). Mechanism of bactericidal activity of silver nitrate—A concentration dependent bi-functional molecule. Braz. J. Microbiol..

[42] Eslami H., Babaei H., Mehrbani S.P., Aghazadeh M., Babaei Z., Nezhad S.K. (2017). Evaluation of antifungal effect of grape seed extract (GSE) on *Candida glabrata* and *Candida krusei*: In vitro study. Biomed. Res..

[43] Simonetti G., Santamaria A.R., D’Auria F.D., Mulinacci N., Innocenti M., Cecchini F., Pericolini E., Gabrielli E., Panella S., Antonacci D. (2014). Evaluation of Anti-Candida Activity of *Vitis vinifera* L. Seed Extracts Obtained from Wine and Table Cultivars. BioMed Res. Inter..

[44] Fraternale D., Ricci D., Verardo G., Gorassini A., Stocchi V., Sestili P. (2015). Activity of *Vitis vinifera* tendrils extract against phytopathogenic fungi. Nat. Prod. Commun..

[45] Nechita A., Filimon R.V., Filimon R.M., Colibaba L.-C., Gherghel D., Damian D., Pașa R., Cotea V.V. (2018). In vitro Antifungal Activity of a New Bioproduct Obtained from Grape Seed Proanthocyanidins on *Botrytis cinerea* Mycelium and Spores. Not. Bot. Horti Agrobot. Cluj Napoca.

[46] Baykan M., Doğan M., Ege D., Kara Z. (2018). Effectiveness of Grape (*Vitis Vinifera,* L.) Seed Extracts on Fungi and Bacteria Management. Selcuk J. Agric. Food Sci..

[47] Sagana M., Rajasekar A., Rajeshkumar S. (2020). Antifungal Activity of Grape Seed Extract Mediated Zinc Oxide Nanoparticles—An In Vitro Study. Plant. Cell Biotechnol. Mol. Biol..

[48] Shivani N., Rajasekar A., Rajeshkumar S. (2020). Antifungal Activity of Grape Seed Extract Mediated Titanium Oxide Nanoparticles against *Candida Albicans*: An In Vitro Study. Plant. Cell Biotechnol. Mol. Biol..

[49] Pingale S.S., Rupanar S.V., Chaskar M.G. (2018). Plant- mediated biosynthesis of silver nanoparticles from Gymnema sylvestre and their use in phtodegradation of Methyl orange dye. J. Water Environ. Nanotechnol..

[50] Bhattacharjee S. (2016). DLS and zeta potential—What they are and what they are not?. J. Control. Release.

[51] Stetefeld J., McKenna S.A., Patel T.R. (2016). Dynamic light scattering: A practical guide and applications in biomedical sciences. Biophys. Rev..

[52] Al-Otibi F., Al-Ahaidib R.A., Alharbi R.I., Al-Otaibi R.M., Albasher G. (2020). Antimicrobial Potential of Biosynthesized Silver Nanoparticles by *Aaronsohnia factorovskyi* Extract. Molecules.

[53] Alotibi F.O., Ashour E.H., Al-Basher G. (2020). Evaluation of the antifungal activity of *Rumex vesicarius* L. and *Ziziphus spina-christi* (L.) *Desf.* Aqueous extracts and assessment of the morphological changes induced to certain myco-phytopathogens. Saudi J. Biol. Sci..

[54] Valand R., Tanna S., Lawson G., Bengtström L. (2020). A review of Fourier Transform Infrared (FTIR) spectroscopy used in food adulteration and authenticity investigations. Food Addit. Contam. Part A.

[55] Balouiri M., Sadiki M., Ibnsouda S.K. (2016). Methods for in vitro evaluating antimicrobial activity: A review. J. Pharm. Anal..

[56] Javan Bakht Dalir S., Djahaniani H., Nabati F., Hekmati M. (2020). Characterization and the evaluation of antimicrobial activities of silver nanoparticles biosynthesized from *Carya illinoinensis* leaf extract. Heliyon.

[57] Daoud A., Malika D., Bakari S., Hfaiedh N., Mnafgui K., Kadri A., Gharsallah N. (2019). Assessment of polyphenol composition, antioxidant and antimicrobial properties of various extracts of Date Palm Pollen (DPP) from two Tunisian cultivars. Arab. J. Chem..

